# The theory of the quantum walk comb laser

**DOI:** 10.1515/nanoph-2024-0768

**Published:** 2025-05-05

**Authors:** Alexander Dikopoltsev, Ina Heckelmann, Barbara Schneider, Mathieu Bertrand, Jérôme Faist

**Affiliations:** Physics Department, 27219 ETH Zurich, Zürich, Switzerland

**Keywords:** laser science, photonic lattices, mode-locked lasers

## Abstract

The development of on-chip optical frequency comb devices paves the way for novel applications in environmental tracking, fast ranging and smart communication solutions. Recently, a new type of frequency comb device, based on a modulated ring quantum cascade laser, was introduced and demonstrated. Here we present a rigorous theoretical study of this type of device, also known as the quantum walk comb laser. We show that resonant phase modulation of a fast gain laser with a dispersive circular cavity is sufficient to support a broadband comb. This method requires the gain to have a sufficiently fast recovery time to support quasi-instantaneous suppression of intensity fluctuations. When this condition is met, the modulation leads to quantum walk dynamics, and then to stabilization onto a stable and controllable frequency comb. We show this type of dynamics through simulations using realistic parameters and reveal the impact of higher-order contributions from gain and dispersion. We also study the resilience of this type of mode-locked laser to noise injection and show its superiority to that of active mode-locking. We believe that this work will allow the development of comb devices with high wall-plug efficiency, arbitrary output spectral shaping and increased stability properties.

## 1 Introduction

Optical frequency comb source miniaturization [1] holds the promise to significantly impact metrology [2], [3], [4], [5], communications [6], [7], [8], and ranging [9], [10]. The first requirement of such sources is to produce spectrally broad light with perfectly spaced frequencies while on-chip. For practical reasons, additional constraints, such as wall plug efficiency and fabrication costs, impact their development [1], [11]. The production of frequency comb devices that fulfill these requirements will introduce an industrial advancement, driving energy efficient sources for dense wavelength communication channels, novel LIDAR schemes and molecular detection applications that can fit in your hand. To make frequency comb sources compact and useful, we are compelled to obtain fine control over the nonlinear processes that govern the broadening of the spectrum.

The mechanisms that generate optical frequency combs are typically divided into a few types, which mainly depend on whether the gain of the laser source and the nonlinear proliferation mechanism of the multiple frequency modes share the same space. From a practical point of view, this difference sets a condition on whether the system can be monolithically fabricated or must use hybrid integration of a laser source and a passive nonlinear chip. With bounds on the strength of nonlinear processes [12], the wall plug efficiencies of such processes differ as well. While integrated devices enjoy highly broadband processes with low loss [11], [13], [14], [15], the monolithic structures benefit from directly pumping the many spectral lines of the frequency comb and reaching overall high power-conversion efficiency [16], [17], [18], [19], [20], [21], [22].

An additional difference lies in the mathematical description of the energy source. This becomes evident in the equation typically used to describe ultrafast optics, the complex Ginzburg–Landau equation (CGLE) [23], which is based on a slowly varying envelope in a copropagating frame with the intracavity light. The difference between monolithic and integrated frequency comb devices is in the power source term in the equation, with either a coherent pump that injects photons with the same frequency and phase, or an incoherent pump that appears as a gain term, typically with saturation that depends on intensity [24]. It has been shown that incoherent pumping terms in a CGLE equation can lead to stabilization in highly excited states, with demonstrations in exciton–polariton cavities [25], [26] or vertical cavity surface emitting lasers [27].

The physical process that gives rise to incoherent pumping is determined by the relative response of the gain saturation [28]. When the response is fast enough, the incoherent pump term is local in the copropagating frame, suppressing fluctuations on very fast time scales. The fast suppression mechanism provides the intracavity light with the properties of a liquid [29], which lead to increased coherent flows, but also the disfavoring of pulses [30]. This is in contrast to the majority of the current techniques to produce frequency combs that are amplitude-modulated, exhibiting pulses in time [14], [31], [32]. For a sufficiently fast incoherent pump, the amplitude is prevented from modulation in time, keeping a quasi-constant intensity, while the phase starts taking on a dynamical role and evolves to produce a frequency comb output [33]. Although the mechanisms that are responsible for the fast processes can vary, the impact is similar – quasi-instantaneous suppression of intensity fluctuations [29]. Only recently, it was shown that such sources can produce an optical frequency comb with perfectly spaced frequencies [16], [30] and constant phase relations that allow for compression of the signal to short pulses [34]. The material platforms that show such formation of frequency-modulated combs span from quantum cascade lasers (QCLs) and interband cascade lasers [16], [17], [35], to quantum dash [20], [21], quantum dot [19], [22] and also quantum well lasers [18], demonstrating the universality of the regime.

Although it is agreed that the mechanism that locks the modes is the incoherent pump term that results from fast gain saturation, only recently the modeling of fast-gain lasers revealed an explanation for mode proliferation [36], [37], [38]. The difficulty here was in identifying the nonlinearities which act to couple frequencies while the intensity of the signal remains constant. For example, a nonlinearity such as the Kerr nonlinearity that depends on the amplitude would only produce an overall trivial phase velocity change that does not broaden the signal, e.g. $i\dot{E}\propto I\left(z\right)E=I_{0}E$ , where *E* is the field, $I\left(z\right)={\left|E\right|}^{2}$ and *I*_0_ is a constant intensity value. Therefore, the required nonlinearity is expected to depend on other quantities, for example the phase of the signal, which is free to vary. Indeed, it was found that in Fabry–Perot semiconductor lasers, the counter-propagating waves interfere to form gain grating (spatial hole burning) that produces four-wave-mixing (FWM) in the form called cross steepening [36], [39]. Effectively, this introduces FWM that depends on the phase of the signal, e.g. $i\dot{E}\propto \phi E$ , where $\phi =\arg\left(E\right)$ , that self-starts a frqeuency comb. However, this spontaneous process naturally lacks control over the final state, while also having an ambiguity in the relative delay inside the optical cycle, which amounts to instabilities and enhanced noise.

In this work, we develop a model for optical frequency comb generation from an incoherently pumped monolithic laser that is modulated to produce highly controllable and stable broadband spectra [40], [41]. We significantly reduced backscattering to avoid competing proliferation processes that lead to instabilities. By resonant modulation of the cavity, we couple the longitudinal modes and generate coherent proliferation and expansion in the frequency domain. At the end of the expansion process, the incoherent pump locks the output onto a broadband state, with a spectral bandwidth that is defined by the modulation depth and cavity dispersion. This method to generate frequency combs presents opportunities for highly stable, controllable and efficient frequency comb sources on a chip.

## 2 Modulated fast-gain ring-laser model

**Figure 1:**
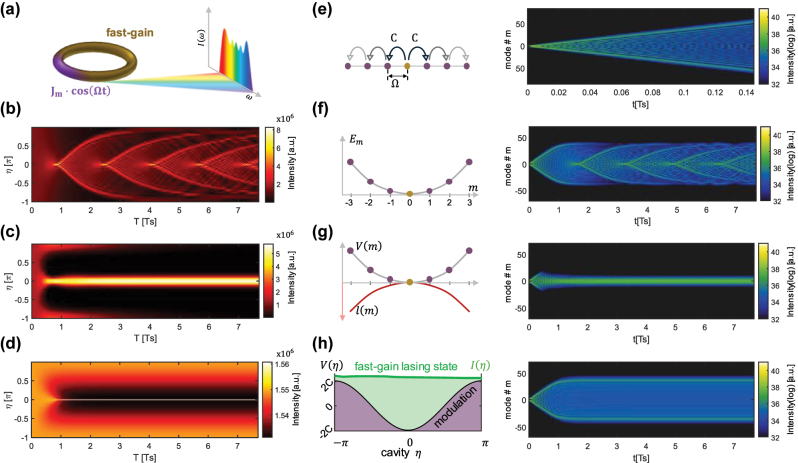
The evolution of the lasing state in an active modulated ring cavity. (a) System schematics of a fast-gain modulated ring laser. The laser is modulated in a section with frequency Ω, which produces mode proliferation. The fast gain is responsible for stabilizing the system so that the emission is coherent and broadband. (b)–(d) Evolution of the field amplitude in the unitless cavity space *η* using Eq. (3), for *β* ≠ 0 in three cases: (b) in the absence of stabilization mechanisms, *I*_*s*_ → ∞, *T*_2_ → 0, where the field develops with uncorrelated dynamics; (c) when gain curvature stabilizes the system, *I*_*s*_ → ∞, *T*_2_ ≠ 0, which results in a narrow signal in time, and (d) when fast-gain is present, i.e. *I*_*s*_ is finite, and the intensity remains quasi-constant and stable. The units of time are normalized to $T_{s}=1/\sqrt{CD}$ , the expansion time of the system. (e)–(g) Lattices describing the coupling and complex potentials in the modal space, and their evolution in the modal space starting from a single mode. (e) Contains only phase modulation, causing coupling, which results in a quantum walk and a ballistic expansion, (f) includes parabolic dispersion, showing uncorrelated dynamics corresponding to (b), and (g) also contains gain curvature, which is a dissipative mechanism that leads to a narrow spectral state, corresponding to (c). While coupling sets the expansion rate, the dispersion *β* dictates a bandwidth limitation. (h) The principle of a modulated cavity with fast gain, forcing a quasi-constant intensity $I\left(\eta \right)$ (left). The signal exhibits the phase modulation as an underlying potential $V\left(\eta \right)$ , with a span of 4*C*. The evolution shows ballistic expansion and then locking onto the broadest available bandwidth by the coupling and dispersion (right), corresponding to (d).

We consider a laser in a ring cavity configuration with circumference *L* and negligible backscattering of the propagating light [42] (Figure 1(a)). We assume there is only one transverse mode allowed, but multiple longitudinal modes that can potentially lase simultaneously. The laser cavity carries losses *α*_*w*_, dispersion *k*_*t*_ and has a saturation intensity *I*_*s*_. When the laser is electrically pumped, the light exhibits a small signal gain of *g*_0_, and a parabolic gain spectral shape with a width *T*_2_ determining a quadratic increase in loss relative to the central frequency. When pumped, the laser supports single-mode operation at the wavelength with the highest gain, which is given by the medium, and thus we define it as the zeroth mode with phase velocity *c*. In the absence of nonlinearities in the material, the single-mode solution is stable, therefore leading to monochromatic lasing.

At time *t* = 0, we start to resonantly modulate the current in a section of the laser, thereby generating a spatiotemporal gain variation of the form $g_{m}\left(x,t\right)=\underset{n}{\sum }a_{n}\cos\left(nKx\right)\cos\left(\Omega t+\phi_{n}\right)$ , where Ω is the modulation frequency, *K* = 2*π*/*L* is the wavenumber of the fundamental mode, and *a*_*n*_ and *ϕ*_*n*_ are the excited amplitudes and phases of the low-frequency cavity modes *n* (Figure 1(a)). In principle, resonant modulation at Ω_*r*_ = *Kc* should produce *a*_1_ ≫ *a*_*n*_ *∀n* > 1. The gain modulation produces an amplitude modulation on top of a quasi-constant signal. When the modulation is deeply close to the threshold and the dynamics of the gain become critical [43], [44], it is possible to reach a pulsed output, a regime we avoid in this study. Moderate modulation alone is inefficient in mode proliferation, leading to negligible intermode coupling. However, the linewidth enhancement factor (LEF) *α* [45], [46] is responsible for additional phase terms that depend on the gain and the amplitude of the modulation. Consequently, the applied amplitude modulation of the gain is converted to phase modulation through the non-zero LEF, resulting in much more efficient side-mode proliferation. We incorporate the modulation in the model by translating the gain variation to a phase modulation of the form $M\left(x,t\right)=\alpha g_{m}\left(x,t\right)$ , as keeping amplitude modulation indeed did not change significantly the result but made the calculation heavier numerically. We also neglect the Kerr nonlinearity added by the LEF as well as higher-order processes induced by the LEF, as the intensity follows mostly the modulated gain signal without substantial intensity variation that can be translated to independent phase dynamics. The master equation of the complex field that describes the dynamics in this system, derived from Maxwell–Bloch equations [36], [37], [47], is then

(1) $$\begin{array}{ll} \frac{1}{c}\frac{\partial E}{\partial t}+\frac{\partial E}{\partial x} & =\frac{1}{2}\left[g_{0}\left(1-I\left(x,t\right)/I_{s}\right)-\alpha_{w}\right]E+i\frac{1}{2}k_{t}\frac{\partial^{2}E}{\partial t^{2}}\\ & \;+\frac{1}{2}g_{0}T_{2}^{2}\frac{\partial^{2}E}{\partial t^{2}}+iM\left(x,t\right)E. \end{array}$$

To describe the evolution of the field in a single coordinate of time, reaching a CGLE form, we shift to a copropagating frame of the optical signal by using *z* = *x* − *ct* and *τ* = *x*/*c*, and set Ω_*r*_ = *Kc*. Using the longitudinal optical modes of the circular cavity, the field in the lab frame is described by $E_{\text{lab}}\left(t,x\right)=e^{i\omega_{o}t-ik_{0}x}\sum A_{m}\left(t\right)e^{im\Omega_{r}t-imKx}$ , and in the copropagating frame of *z* and *τ* by $E\left(z,\tau \right)=\sum A_{m}\left(\tau \right)e^{-imKz}$ , where *A*_*m*_ are the slowly varying amplitudes of modes with resonant optical frequencies. The modulation term takes the form

(2) $$\begin{array}{ll} M\left(z,\tau \right) & =\frac{\alpha }{2}\underset{n}{\sum }a_{n}\left[\cos\left(\left(n\Omega_{r}+\Omega \right)\tau -z\Omega /c+\phi_{n}\right)\right.\\ & \;\left.+\cos\left(\left(n\Omega_{r}-\Omega \right)\tau +z\Omega /c-\phi_{n}\right)\right]. \end{array}$$

In this case, the coupling between the modes will require a phase-matching term of the form ∼*e* ^*ilKz*^, with *l* ∈ Z . Therefore, for near-resonant modulation, Ω = Ω_*r*_ + ΔΩ, with detuning ΔΩ ≪Ω, and under a rotating wave approximation, the modulation term is reduced to $M_{e}\left(z,\tau \right)=\frac{\alpha }{2}a_{1}\cos\left(Kz-\Delta \Omega \tau -\phi_{1}\right)$ . Notice that we have assumed $\cos\left(Kz+\frac{\Delta \Omega z}{c}\right)\approx \cos\left(Kz\right)$ , due to the assumption that $\frac{\Delta \Omega }{Kc}\ll 1$ . The resulting equation takes the following CGLE form

(3) $$\begin{array}{ll} \frac{\partial E}{\partial \tau } & =\frac{1}{2}c\left[g_{0}\left(1-I/I_{s}\right)-\alpha_{w}\right]E+i\frac{1}{2}\beta K^{2}\frac{\partial^{2}E}{\partial \eta^{2}}\\ & \;+\frac{1}{2}g_{c}K^{2}\frac{\partial^{2}E}{\partial \eta^{2}}+icM_{e}\left(\eta ,\tau \right)E, \end{array}$$

where used dispersion as *β* = *k*_*t*_ *c* ^3^, the gain curvature $g_{c}=g_{0}T_{2}^{2}c^{3}$ , and the corotating coordinate *η* = *Kz* − ΔΩ*τ*. Figure 1(b)–(d) show the evolution over time *τ* in space *η* of the state $E\left(\eta ,\tau \right)$ , starting from a single mode *m* = 0 for three different cases while *β* ≠ 0. For the simulation in this work, we have used realistic parameters, given in Appendix D. When *I*_*s*_ → ∞ and *T*_2_ → 0 (Figure 1(b)), the equation is linear and behaves like a Hermitian system but lacks a stabilization mechanism. The dephasing of the eigenmodes excited by the initial state leads to uncorrelated dynamics. When *T*_2_ is finite (Figure 1(c)), the dissipative gain curvature, *g*_*c*_ *K* ^2^, acts as a stabilization mechanism, and together with the modulation *M*_*e*_, induces a gaussian state in time, as predicted in active mode-locking processes [48]. Finally, when *I*_*s*_ is finite (Figure 1(d)), the fast-gain acts as the main stabilization mechanism and maintains a quasi-constant intensity state. Although the picture in the cavity space provides signatures of stability, the evolution of such a system is more intuitively described in the frequency domain. Specifically, as the modulation introduces direct coupling between longitudinal modes, in the following we will analyze the quantum walk comb laser dynamics through a modal space picture. We note that the modes are almost equally spaced and nearest neighbors are coupled by modulation, which can be mapped to a “tight binding” model of a lattice in a synthetic frequency space.

## 3 Active photonic lattice in a synthetic dimension

To provide intuition for the dynamics of Eq. (3), we consider the mode coupling that *M*_*e*_ introduces. Therefore, we will now describe the system from the point of view of a lattice in the frequency domain, which we call a synthetic dimension. We use this terminology, as this is related to a whole field of light manipulation in linear systems [49], and that has the potential to revolutionize mode-locked sources [50]. We ascribe a lattice location index *m* to frequency *f*_0_ + *mf*, and equivalently through a discrete Fourier transform, we derive a set of equations for the linear part of the modes’ evolution using the aforementioned ansatz for $E\left(z,\tau \right)$ :

(4) $$\begin{array}{ll} i{\dot{A}}_{m} & =\frac{1}{2}\beta m^{2}K^{2}A_{m}-i\frac{1}{2}g_{c}m^{2}K^{2}A_{m}\\ & \;+\frac{1}{4}c\alpha a_{1}\left(A_{m+1}e^{-i\Delta \tau }+A_{m-1}e^{i\Delta \tau }\right), \end{array}$$

where *a*_1_ is the relevant modulation amplitude defined above, and *z* coordinate is shifted to nullify *ϕ*_1_ and keep the sign of the coupling positive, so that $z\to z-\left(\phi_{1}+\pi \right)/K$ . We use $D=\frac{1}{2}\beta K^{2}$ , $G_{c}=\frac{1}{2}g_{c}K^{2}$ , $C=\frac{1}{4}c\alpha a_{1}$ , and the ansatz *A*_*m*_ = *B*_*m*_ *e* ^*i*Δ*mτ*^ to get

(5) $$i{\dot{B}}_{m}=Dm^{2}B_{m}+\Delta mB_{m}+i(G_{0}-G_{c}m^{2})B_{m}+C\left(B_{m+1}+B_{m-1}\right),$$

which describes linear dynamics in the modal space with coupling *C* (Figure 1(e), left), an effective quadratic potential *Dm* ^2^ (Figure 1(f), left) with a linear bias Δ*m*, gain *G*_0_ = (g_0_-α_w_)c/2, and quadratic loss *G*_*c*_ *m* ^2^ (Figure 1(g), left). The following present dynamics that depend on the different elements of this system. In Figure 1(e)–(g), we present the dynamics in the unbiased modal space lattice, i.e. Δ = 0, with *D* = *G*_*c*_ = 0, *D* ≠ 0, *G*_*c*_ = 0 and *D* ≠ 0, *G*_*c*_ ≠ 0, respectively, where Figure 1(f) and (g) correspond to Figure 1(c) and (d). Figure 1(e) shows the well-known pattern of a quantum walk [51], [52], [53], [54], [55], the quantum analogue of the random walk, where the traveling particle also has phase. In this case the intensity remains flat, $E\left(\eta ,\tau \right)=E_{0}$ , throughout the whole evolution. However, when dispersion is present (Figure 1(f)), *D* ≠ 0, we observe a bound on the maximally accessible frequency bandwidth and dephasing of the state. When gain curvature is added (Figure 1(g)), *G*_*c*_ ≠ 0, the state locks onto a Gaussian state, much narrower than the limit set by the dispersion. Gain curvature causes the state to narrow down in the modal space and a strong amplitude change in the real space (Figure 1(c)).

We can now add the nonlinear term of the gain saturation to the equation. This term is represented as a local operator in the real space, clamping the intensity to a certain value, i.e. $I\left(\eta \right)∼I_{0}$ , and suppressing intensity fluctuations faster than all other timescales (Figure 1(h), left). We note that the modulation occurs in the same effective space, *η*, generating an effective potential $V\left(\eta ,\tau \right)\propto M_{e}\left(\eta ,\tau \right)$ . However, in the modal space, the gain saturation becomes a long-range dissipative term, which we describe using the following form

(6) $$i\dot{\vec{B}}=H\vec{B}-i\gamma {\vec{F}}_{NL}\left(\vec{B}\right),$$

where we define *H* as the linear operator of the right-hand side of Eq. (5), and $\gamma =\frac{1}{2}g_{0}c/I_{s}$ . Here, the fast gain saturation term ${\vec{F}}_{NL}=EE^{*}E$ is of a four wave mixing nature [30], [56], [57], and can be written as $F_{NL,m}=\underset{\mathrm{jpl}}{\sum }\delta_{j+l-m-p}B_{j}B_{l}B_{p}^{*}$ (derivation shown in Appendix E). The evolution in this lattice, in the synthetic frequency space, i.e. when *I_s_* is finite, is presented in Figure 1(h) (right), which shows an initial quantum walk and subsequent stabilization onto a broad state. We observe that a system with fast gain, even in the presence of gain curvature, recovers the original bandwidth set by the passive system. Interestingly, in the absence of dispersion and gain curvature, i.e. a confining potential, the propagation for either fast or slow gain would be identical. In the following, we will derive the limits in the spectral domain.

## 4 Eigenstates of the frequency lattice

We will now use the modal space description to derive the available stationary states of the system. As mentioned, the coupling terms introduce coherent lattice dynamics of a quantum walk [51], [52], [53], [54], [55] (Figure 1(e)). In contrast to diffusive expansion, the unique feature of the quantum walk dynamics, which results from interference, is its fast ballistic expansion [58], [59]. However, introducing *D* ≠ 0 will change this dynamic by breaking the translational symmetry and modifying the quantum walk dynamics (Figure 1(f)). This is restricting the analysis of this system from momentum and energy consideration in band structures to solely eigenmodes and eigenvalues.

**Figure 2:**
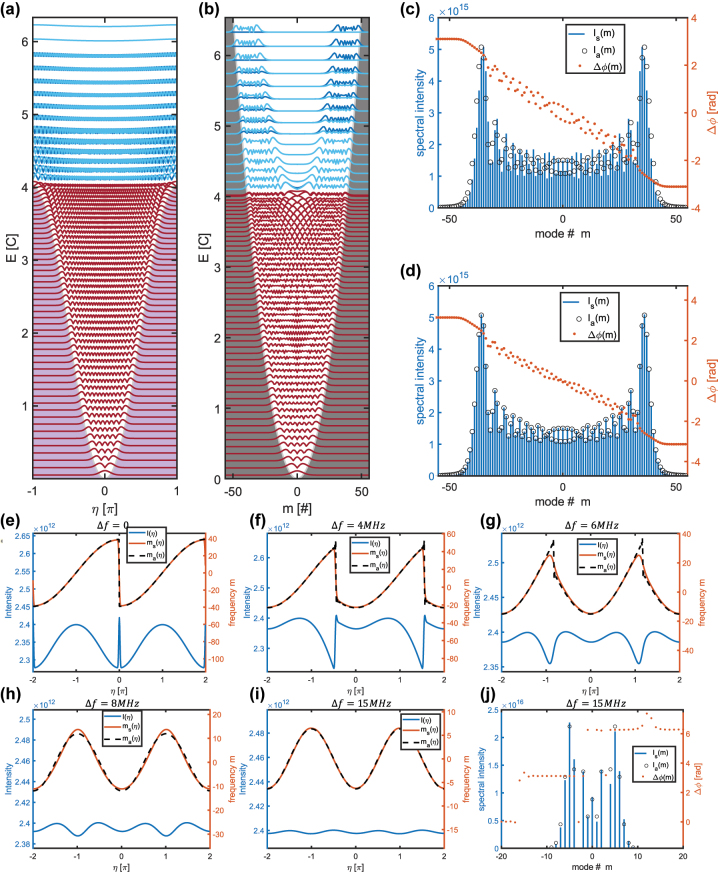
Linear eigenmodes and nonlinear steady states. (a) And (b) are eigenmodes of Eq. (5) in the cavity space and modal space, respectively. The eigenmodes are trapped in the quadratic potential, with an upper limit on the maximal available trapped mode. This limit is set by the available kinetic energy in the system, 4*C*. (c) And (d) are steady state spectra calculated from simulation for two values of gain curvature, *g*_*c*_ = 2 and *g*_*c*_ = 0.2, respectively, presented with the analytical solution. They exhibit a Hermite–Gaussian intensity distribution (blue) with linear intermodal phase difference Δ*ϕ* (red). The simulated amplitudes *I*_sim_(*m*) and the analytical solution *I*_*a*_(*m*) agree well, especially for reduced *g*_*c*_. (e)–(i) Steady-state instantaneous frequency taken from simulations and compared to the analytical solution for different detuning values above and below the critical detuning Δ*f*_*c*_ = 6.93 MHz. We also present the small amplitude variation, *f*_*A*_, occurring due to gain curvature. (j) Steady state spectrum for off-resonant modulation with Δ*f* > Δ*f*_*c*_, derived by simulations and compared to the analytical solutions. Here the phase difference between the modes has a 0 or *π* difference, typical to electro-optically modulated combs.

The effective set of equations required to find the eigenmodes are of the form *ϵB*_*m*_ = *Dm* ^2^ *B*_*m*_ + *CB*_*m*+1_ + *CB*_*m*−1_, with *ϵ* being the eigenvalue (in the case of *G*_*c*_ = Δ = 0). The first term is a quadratic potential that depends on the synthetic space coordinate *m*, $V\left(m\right)=Dm^{2}$ , and the mode coupling is a discrete second derivative equivalent to the kinetic energy of the state. These coupling terms can only carry a finite amount of kinetic energy equal to 4*C*, given by the coupling energy span. This set of equations can be then treated as a discrete version of a quantum harmonic oscillator, where the solutions of this system will have Hermite–Gauss nature for a finite range of energies restricted by the available kinetic energy, 4*C*. Above this value, the system behaves differently, having the modes bound to the local potential, like Wannier-stark state that are bound without a potential well [29]. Figure 2(a) and (b) show the eigenmodes of such a system for Δ = *G*_*c*_ = 0. The broadest trapped state utilizes the maximum kinetic energy, therefore $Dm_{\max}^{2}=4C$ , leading to $m_{\max}=2\sqrt{C/D}$ , which sets a spectral bandwidth limit of *W*_max_ = 2f_*r*_ *m*_max_, where *f*_*r*_ = Ω_*r*_/2*π* is the resonance frequency of the laser cavity. We note that, in contrast to Fourier relations, due to the duality between the two conjugate coordinates in harmonic oscillators, the solutions follow a positive relation between the two spaces with respect to width, i.e., the narrow solutions in frequency are also the narrow solutions in time, while the broad solutions in frequency are also the broad solutions in time. The lowest supermode carries a bandwidth of $W_{1}=2\cdot \sqrt[{4}]{C/D}f_{r}$ , while the broadest supermode trapped in the quadratic potential carries a bandwidth of $W_{\max}=2\cdot \sqrt[{4}]{C/D}W_{1}$ . Considering a finite detuning from resonance ΔΩ, the potential becomes $V\left(m\right)=Dm^{2}+\Delta \Omega m$ . As an effect, the central mode *m* = 0 is shifted from the minimum of the potential by $m_{0}=\frac{\Delta \Omega }{2D}$ , and its minimum is reduced by $E_{\Delta }=\frac{\Delta \Omega^{2}}{4D}$ , compared to the resonant case ΔΩ = 0. When the available kinetic energy is insufficient to support trapped states, the available states will become localized to the local energy and have the shape of a Wannier-Stark state. This happens for *E*_Δ_ above the kinetic energy limit, which occurs above a critical detuning $\Delta \Omega_{c}=\sqrt{8CD}$ . This sets a transition between two ranges of RF detuning, the resonant and off-resonant modulation regimes.

We can now analyze the dynamics and steady states in this system using the eigenmodes that we have found. Figure 1(f) shows a simulation of the time evolution in this system, initially expanding ballistically but then reaching a limit set by the quadratic potential and the kinetic energy, *m*_max_. Once this limit is reached, the expansion stops, and mode dephasing effects appear due to additional contributions from the quadratic potential. To study a realistic laser system, we then introduce gain curvature by setting *G*_*c*_ ≠ 0, which introduces a non-Hermitian modal-space dependent contribution. In this case, we consider gain saturation acting on the average intensity in the cavity $\left\langle I,\left(z\right)\right\rangle$ of the form $g_{0}\left(1-\left\langle I,\left(z\right)\right\rangle /I_{s}\right)$ (equivalent to *I*_*s*_ → ∞). This slow gain saturation effectively normalizes the overall power without modifying the temporal pulse shape. In this linear system, it is easy to show that only the eigenmode with the maximal overlap with the gain will have an infinite lifetime. Figure 1(g) shows the evolution in time, where the initial quantum walk from a single mode dissipates into the first Hermite–Gauss mode with a Gaussian envelope both in frequency and time, i.e. a pulse of spectral bandwidth *W*_1_ [48]. This also resembles a condensation process [60], where the system thermalizes to the lowest energy state. Although this stabilization process is beneficial for pulse generation, for many applications, frequency comb sources are required to have broadband spectra, which is not optimized here. It would be beneficial to utilize the broadest available bandwidth set by the limit *W*_max_, but in the discussed slow-gain case, the required spectrally and temporally delocalized supermodes are inaccessible [48]. The method of driven mode-locking could greatly benefit from a different approach, which prevents the state from condensing to the lowest energy, and therefore the lowest available bandwidth *W*_1_.

## 5 Synthetic dimension dynamics in fast-gain lasers: quantum walk comb laser

As seen in the previous section, resonant modulation of a laser system alone does not ensure the generation of broadband combs, since dissipation leads to destabilization of spectrally delocalized supermodes and thus a contraction of the spectrum. Our system, however, contains an additional term to the CGLE that stems from the fast dynamics of carriers in semiconductor lasers. When the gain recovery time is fast, it can support quasi-instantaneous saturation of the form $g_{0}\left(1-I\left(z\right)/I_{s}\right)$ , effectively forcing constant intensity all around the laser cavity. We note that the fast-gain is the same property that locks the modes proliferated by cross-steepening in Fabry–Perot lasers, which requires bi-directional lasing [30], [36], [37]. When the fast gain term is considered, the only allowed dynamics in the laser cavity is given by its phase, effectively changing the focus from the photon density ${\left|E\right|}^{2}$ , which remains constant, to the photonic current density $j\propto \beta Im\left\{E^{*}\frac{\partial E}{\partial x}\right\}∼\beta \frac{\partial \phi }{\partial x}{\left|E\right|}^{2}$ , which is also proportional to the instantaneous frequency of the field, $f_{i}=c/2\pi \frac{\partial \phi }{\partial x}$ . This type of field in fast-gain lasers can carry many frequencies while maintaining a quasi-constant intensity, extended over the whole cavity. To maximize the duration of the state in time, fast-gain leads to a different stabilization process. This means that the system opposes stabilization on short forms of fields in time like pulses. On the contrary, fast-gain lasers select the broadest available modes in time to construct the final state, like the high-order Hermite–Gauss modes we found above. As a result, instead of collapsing to the narrow Gaussian mode of bandwidth *W*_1_, fast-gain lasers under resonant modulation support the generation of quantum walk comb laser states, i.e. they stabilize on states with the broadest available frequency bandwidth *W*_max_ ≫ *W*_1_ in the system, which is highly useful for OFC applications (Figure 2(c) and (d)).

## 6 Analytical derivation of the steady-state

To find the steady states in Eq. (3), we turn back to the effective space description and propose a solution of the following form, assuming an eigenfrequency Δ*ω*, while the spatial field is copropagating with the modulation:

(7) $$E=A_{0}\left(1-f\left(\eta \right)\right)\exp\left(i\phi \left(\eta \right)+i\Delta \omega \tau \right).$$

Here *f* is a small variation of the amplitude, so that *f* ≪ 1. By substituting *E* into Eq. (3), we can find the following set of coupled equations for the real and imaginary parts (derivation in Appendix A):

(8) $$\begin{array}{ll} \Delta \omega -\phi^{\prime }\Delta \Omega & =-D\frac{f^{\prime \prime }}{\left(1-f\right)}-G_{c}\frac{2if^{\prime }}{\left(1-f\right)}\phi^{\prime }\\ & \;+G_{c}\phi^{\prime \prime }-D\phi^{\prime 2}+2Ccos\left(\eta \right), \end{array}$$

(9) $$\begin{array}{ll} \frac{f^{\prime }}{\left(1-f\right)}\Delta \Omega & =\frac{1}{2}c\left[g_{0}\left(1-\frac{A_{0}^{2}{\left(1-f\right)}^{2}}{I_{\text{sat}}}\right)-\alpha_{w}\right]-G_{c}\frac{f^{\prime \prime }}{\left(1-f\right)}\\ & \;+D\frac{2if^{\prime }}{\left(1-f\right)}\phi^{\prime }-D\phi^{\prime \prime }-G_{c}\phi^{\prime 2}. \end{array}$$

Due to the suppression of fluctuations and the expected small intensity variation, we look for solutions with *f*, *f*′, *f*″ → 0 (later justified). For the first-order solution, we solve Eq. (8) for the phase. We neglect the contributions of the gain curvature by taking *G*_*c*_ *ϕ*″ → 0, which is justified in Appendix B. The condition on the imaginary part turns to

(10) $$\phi^{\prime }=\frac{\Delta \Omega \pm \sqrt{\Delta \Omega^{2}-4D\Delta \omega +8CDcos\left(\eta \right)}}{2D}.$$

At this point, we do not know the overall eigenfrequency Δ*ω*, and therefore we can find a continuum of states to match this problem. However, we are looking for a single continuous solution where the frequencies have both positive and negative values. Such a solution must use the two branches of *ϕ*′, which would be stitched at the point *η* where the square root is zero, *η*_*s*_, and fulfills $\Delta \Omega^{2}-4D\Delta \omega +8CDcos\left(\eta_{s}\right)\to 0$ . To keep also a finite derivative ${\left(\phi^{\prime }\right)}^{\prime }$ at this point, we must also require that

(11) $$\phi^{\prime \prime }=\frac{\mp 2Csin\left(\eta_{s}\right)}{\sqrt{\Delta \Omega^{2}-4D\Delta \omega +8CDcos\left(\eta_{s}\right)}}<\infty .$$

The stitching points, at which the denominator goes to zero, also require that $\sin\left(\eta_{s}\right)\to 0$ , which sets *η*_*s*_ = *π* as this point should provide the lowest value of the cosine function for the square root to be real (excluding *η*_*s*_ = 0). In this case, the eigenfrequency is set to $\Delta \omega =\frac{\Delta \Omega^{2}}{4D}-2C$ . We plug this value back into the equation and have

(12) $$\phi^{\prime }=\frac{\Delta \Omega }{2D}\pm 2\sqrt{\frac{C}{D}}\cos\left(\frac{\eta }{2}\right).$$

We integrate the solution *ϕ*′ to find the phase

(13) $$\phi \left(\eta \right)=\frac{\Delta \Omega }{2D}\eta \pm 4\sqrt{\frac{C}{D}}\sin\left(\frac{\eta }{2}\right)+\phi_{0}.$$

The solution *ϕ* is correct in the whole range of *η* values only when boundary conditions do not apply. Although the system is periodic, the periodic boundary conditions set by the cavity length do not necessarily match the periodicity of the above form. To find the correct solution, we look for a range of this solution where the overall phase is periodic, meaning $\phi \left(\eta_{0}\right)=\phi \left(\eta_{0}+2\pi \right)$ . We apply the periodic boundary conditions to *ϕ*, which results in

(14) $$\eta_{0}=2\text{arcsin}\left(\mp \frac{\Delta \Omega }{\sqrt{CD}}\frac{\pi }{8}\right),$$

and depends on the offset of the modulation frequency ΔΩ. Then, the constructed periodic solution will have the form $\phi_{s}\left(\eta \right)=\phi \left(\eta \right)\forall \eta_{0}⩽\eta <\eta_{0}+2\pi$ , where $\phi_{s}\left(\eta \right)=\phi_{s}\left(\eta +2\pi \right)$ . Figure 2(c) and (d) show a comparison of the steady-state spectral amplitude and phase differences between simulations of Eq. (3) and the analytical solution from Eq. (12), for two different values of gain curvature. Here, it is clear that the state is not a Bessel function, as the intermode phases are not spaced by only 0 or *π*. In fact, we observe that the sum of these differences adds to 2*π*, which is a beneficial relation for frequency-modulated combs that span over the whole cycle. The spectral amplitude is compared to the analytical solution. When gain curvature is present, the simulation and analytical solutions slightly differ, but with decreasing gain curvature, the correspondence becomes better. In the spatial domain, the solution is mainly defined by its instantaneous frequency. Figure 2(e)–(g) show the value of the instantaneous frequency found by simulations and the analytical solution. These are in good agreement, where the deviation increases for detuning closer to the critical value ΔΩ~ΔΩ_c,_ specifically at the instantaneous frequency discontinuity. This occurs due to incomplete assumptions for the solutions at this critical value of detuning.

### 6.1 Bandwidth at near-resonant modulation

To find the bandwidth of the state when $\left|\Delta \Omega \right|<\Delta \Omega_{c}$ , we look for the largest available phase derivative span in the signal, with a stitching point at *η*_0_. We expect the global extrema to appear either at *ϕ*″ = 0, or at the boundaries, where the extrema values are $\phi_{\text{extrema}}^{\prime }=\Delta \Omega /2D\pm 2\sqrt{C/D}$ . At the edges we expect

(15) $$\begin{array}{ll} \phi_{\text{edges}}^{\prime } & =\frac{\Delta \Omega }{2D}\pm 2\sqrt{\frac{C}{D}\left(1-{\left(\frac{\Delta \Omega }{\sqrt{CD}}\frac{\pi }{8}\right)}^{2}\right)}, \end{array}$$

where we used the relation cos(asin(±x))= $\sqrt{\left(1-{\text{x}}^{2}\right)}$ . As *ϕ*′ it truncated with half a period of a cosine, we expect only a single local extremum to be included, for example the positive one, which would be naturally larger than the positive edge extremum. The lowest *ϕ*′ value will be then at the negative edge value, while the negative local extremum is outside the solution. We conclude that the overall bandwidth is limited on one side by a local extremum and the other by an edge, leaving a bandwidth of

(16) $$\begin{array}{ll} W_{\Delta \Omega } & =\frac{cK}{2\pi }\left|\phi_{\text{extremum}}^{\prime }-\phi_{\text{edge}}^{\prime }\right|\\ & =2f_{r}\sqrt{\frac{C}{D}}\left(1+\sqrt{1-{\left(\frac{\Delta \Omega }{\sqrt{CD}}\frac{\pi }{8}\right)}^{2}}\right). \end{array}$$

At resonant modulation for ΔΩ = 0, the bandwidth is *W*_ΔΩ=0_ = 4*f*_*r*_ √(C/D)= *W*_max_, as expected from the energy consideration in the synthetic space.

### 6.2 Amplitude deviation

Although we neglected the amplitude variation to find the phase, there is a direct impact of the phase on the amplitude. We derive this impact on the amplitude shape when modulation is in the resonant region. We keep *G*_*c*_ → 0, so that the equation for the real part is then (justified in Appendix B)

(17) $$D\phi^{\prime \prime }=\frac{1}{2}c\left[g_{0}\left(1-\frac{A_{0}^{2}{\left(1-f_{A}\right)}^{2}}{I_{\text{sat}}}\right)-\alpha_{w}\right].$$

From this formula, we can derive the steady-state amplitude (Appendix C) and find that

(18) $$f_{A}\approx \frac{D}{c\left(g_{0}-\alpha_{W}\right)}\phi^{\prime \prime },$$

where the amplitude is directly impacted by the variations in the phase. Figure 2(e)–(i) compares the instantaneous frequency, $m\left(\eta \right)$ , which is proportional to *ϕ*′, to the amplitude variation, *f*_*A*_. This solution results in a maximal amplitude variation of $f_{A,\max}=\frac{2\sqrt{DC}}{c\left(g_{0}-\alpha_{W}\right)}∼10^{-3}\ll 1$ , which verifies our initial assumption for this range of values.

## 7 Strong detuning regime

As we observe in the lattice model, detuning imposes an offset of the minimum of the dispersion-related potential relative to the point of highest gain. When this offset is sufficiently large, meaning ΔΩ > ΔΩ_c_, the initial conditions and gain curvature set a different stable nonlinear solution to the system. To find this family of solutions, we assume that the detuning is large enough, so that we could use the approximation $\left|\Delta \Omega \phi^{\prime }\right|\gg \left|D\phi^{\prime 2}\right|$ and write the phase equation as

(19) $$\frac{G_{c}}{\Delta \Omega }\phi^{\prime \prime }+\phi^{\prime }=\Delta \omega -\frac{2C}{\Delta \Omega }\cos\left(\eta \right),$$

where the solution of this driven linear ordinary differential equation is

(20) $$\phi^{\prime }=\frac{\Delta \omega }{\Delta \Omega }-\frac{2C}{\Delta \Omega \sqrt{{\left(\frac{G_{c}}{\Delta \Omega }\right)}^{2}+1}}\cos\left(\eta +atan\left(\frac{G_{c}}{\Delta \Omega }\right)\right).$$

Here, as previously, Δ*ω* is set by the gain curvature that chooses the state with the higher overlap with the central frequencies, i.e. Δ*ω* = 0. The instantaneous frequency and spectra associated with this regime are shown in Figure 2(h)–(j), where these states are cosine shaped in their instantaneous frequency and consequently Bessel-shaped with a symmetric spectrum. We notice that in this regime, the phase is following directly the shape of the modulation, as would be in the case of linear phase modulation by an external EO modulator. From the point of view of modulation overlap, this process is resonant and occurs inside the laser, being more similar in its nature to a frequency comb regime found in actively mode-locked lasers when the modulation is strongly detuned. In practice, the stabilization processes are completely different. Here, the gain recovery time is fast, and the suppression of fluctuations leads to higher stability. We will analyze this stability in Section 8.

**Figure 3:**
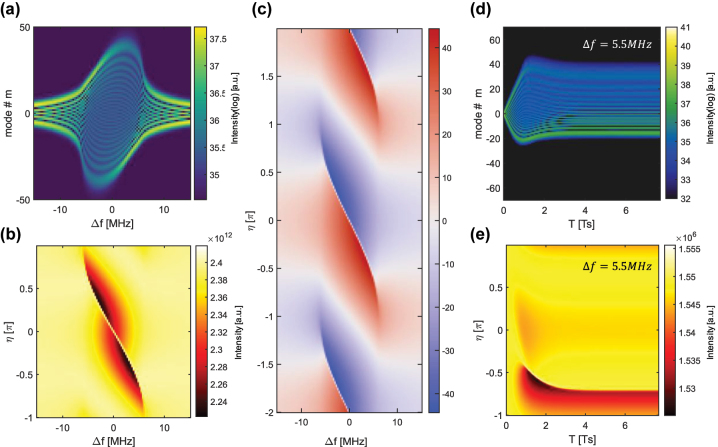
Steady states in the quantum walk comb laser versus RF detuning in simulations. (a) Steady state spectrum as a function of RF detuning Δ*f*. (b) Intensity in the cavity versus RF detuning Δ*f*. (c) Two periods in ***η*** of the instantaneous frequency in units of *m* as a function of the detuning frequency Δ*f*. (d) and (e) Time evolution under significant detuning Δ*f* ∼ ΔΩ_*c*_/2*π* of a state initialized at *m* = 0. The evolution shows an overdamped oscillation and then stabilization into an asymmetric spectrum.

Although we have a general description of the analytical solutions in both the on- and off-resonant regime, it is imperative to acquire their spectral shape from the complete and non-approximated CGLE (Eq. (3)). Figure 3(a)–(c) show the value of the spectral intensity, and amplitude and instantaneous frequencies in simulations for a range of detuning values, Δ*f* = ΔΩ/2π. Although the spectral shape in Figure 3(a) is smoothly deforming in the whole range, Figure 3(b) and (c) present an abrupt transition between two regimes. When $\left|\Delta \Omega \right|<\Delta \Omega_{c}$ , the regime is resonant and follows a solution of the form presented in Eq. (13), and for all other values the steady state follows the solution in Eq. (20). The two regimes are also related to the two groups of eigenmodes presented in Figure 2(a) and (b), the trapped Hermite–Gaussians (red) and the Wannier–Stark like modes above the maximum kinetic energy (blue). Figure 3(d) and (e) show the time evolution of a state that was initiated with a single mode at *m* = 0, in frequency and cavity amplitude, respectively, at a detuning value ΔΩ ∼ ΔΩ_*c*_. We observe an initial oscillation and then stabilization on a steady spectrum. This type of oscillatory dynamics is experimentally studied in a different work [29].

## 8 Effects of significant gain curvature and third order dispersion

**Figure 4:**
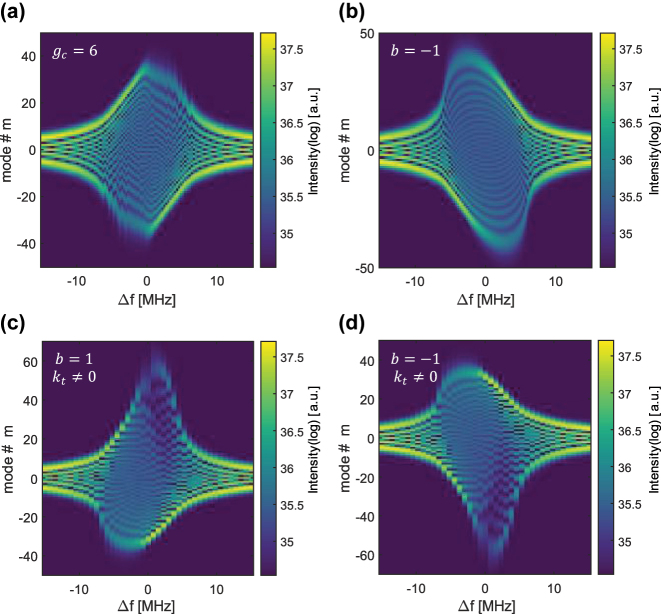
Spectra impacted by gain curvature, sign of dispersion, and third-order dispersion. (a)–(d) Spectra versus RF detuning, Δ*f*, based on the parameters used in Figure 3(a). In (a) the gain curvature is three times larger, which for this value presents a strong limitation on the spectral shape. In (b), the sign of the dispersion is flipped, therefore the spectrum is mirrored symmetrically around *m* = 0. (c) and (d) Have third order dispersion, positive and negative, respectively. The third-order dispersion deforms the spectral shape and breaks the previous symmetry of flipping both detuning and the modal axis.

Although, at first order, gain curvature and third-order dispersion are less significant than quadratic dispersion, modulation and fast gain, these impact the shape of the spectrum. To study the impact of gain curvature and third-order dispersion on the steady state, we perform simulations to find the dependence of the spectra at different detuning values on the gain curvature effective parameter, *G*_*c*_, and third order dispersion parameter $D_{3}=\frac{1}{6}k_{t,3}c^{4}K^{3}$ , where *k*_*t*,3_ is the third-order dispersion is in units of $\frac{fs^{3}}{mm}$ . Figure 4(a) displays the spectrum as a function of detuning, with the same parameters as in Figures 2 and 3, but with *g*_*c*_ = 6, 3 times larger. It is clear that the spectrum is limited by a condition related to gain curvature on top of the maximum bandwidth limit that is set by only modulation and dispersion. Figure 4(b) shows the spectra with the same original conditions, but where dispersion has the opposite sign. This also flips the sign of the instantaneous frequency and reflects the spectrum symmetrically around *m* = 0. Figure 4(c) and (d) show the impact of third-order dispersion on the spectra (*k*_*t*3_ ≠ 0) as a function of injection detuning. We observe that the symmetry between positive and negative detuning is broken, depending on the sign of the third-order dispersion.

## 9 Noise and stability

**Figure 5:**
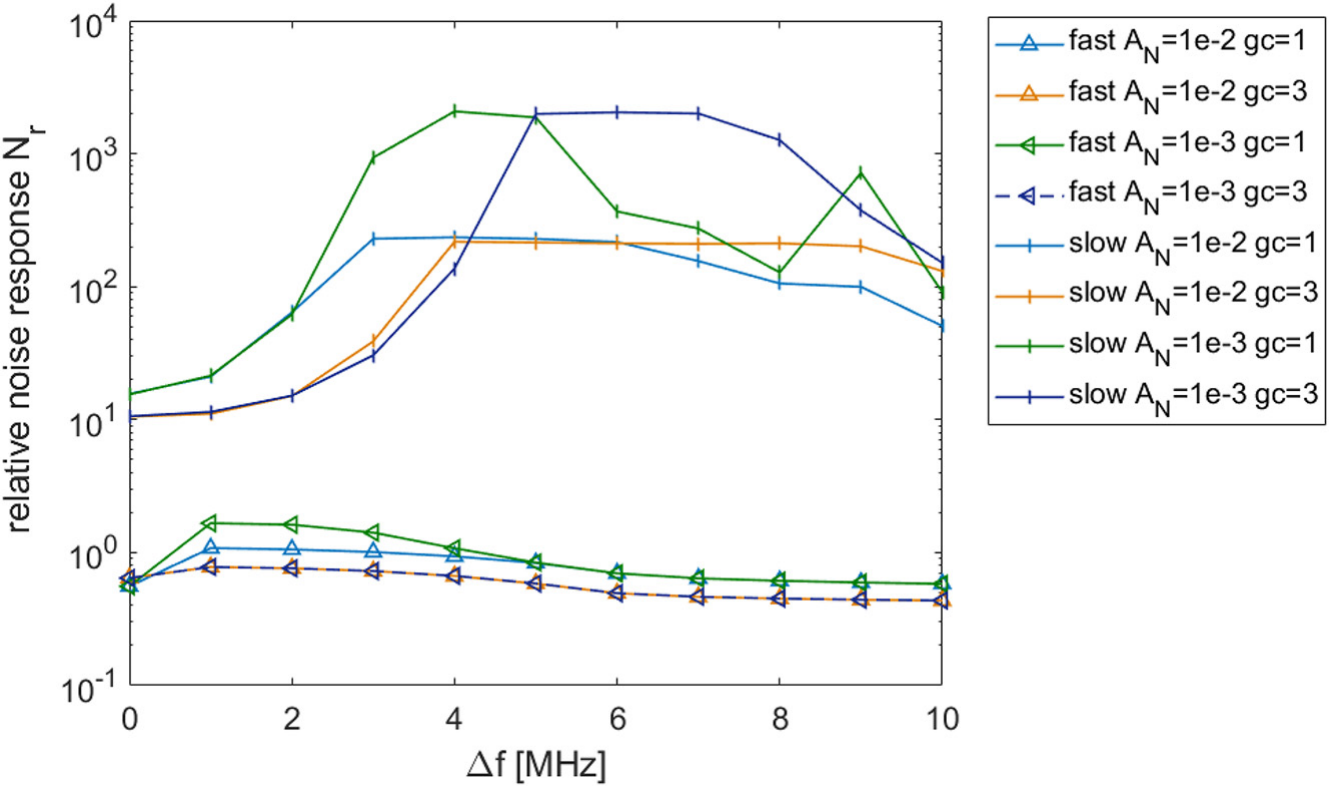
Intensity noise level for slow and fast gain. The relative noise response is the square root of the intensity variance normalized by the total intensity and noise generation rate. The noise is presented in logscale and shows the supremacy of fast gain lasers in terms of noise for the whole range of frequencies for various values of gain curvature and noise generation rates.

Lasers with fast gain that stabilize through gain saturation have noise suppression properties that differ from dissipative slow gain systems. It was shown through an analysis of fluctuations [61], [62], [63], that noise that does not match the phase of the underlying nonlinear steady state in the specific point where it is located will be quickly suppressed like in a single mode laser. Therefore, stability would follow approximately a Shallow-Townes limit [64]. This is in contrast to lasers with slow gain that suppress intensity fluctuations on average, with the form $g_{0}\left(1-\left\langle I,\left(z\right)\right\rangle /I_{s}\right)$ , letting the state destabilize from its self-preserving shape. Figure 5 shows calculations of noise added to a system with fast and slow gain in various regimes. We model the noise as an additional term on the right-hand side of Eq. (3), in the following form $A_{N}\sqrt{I_{0}}\left(X\left(z\right)+iY\left(z\right)\right)/T_{c}$ , where $X,Y\in \left[-1,1\right]$ are random with a uniform distribution, $I_{0}=I_{s}\left(1-\alpha /g_{0}\right)$ is the steady state intensity and *A*_*N*_ is a variable amplitude of the noise. In the absence of noise, we reach a steady state of the system and use the noise term above to perturb it. We then calculate the variance of the intensity $\sigma_{I}\left(z\right)$ relative to the steady state for 400,000 cycles. Every point in Figure 5 is calculated as $N_{r}=\sqrt{\sum \sigma_{I}^{2}\left(z\right)/N_{l}}/2I_{0}A_{N}$ , which is exactly *A*_*N*_ for a signal that contains only the noise input, and where *N*_*l*_ is the number of points used for the cavity space. When the detuning, Δ*f*, is small, there is more than an order of magnitude difference in the response of the fast gain compared to the slow gain. The power fluctuation levels for the fast gain show significantly lower amplitudes than for slow gain. Further away from resonance, this becomes even more drastic, reaching a difference of 2–3 orders of magnitude. This showcases the improved noise properties of the quantum walk comb laser compared to regular active mode locking.

## 10 Discussion

In this work, we have derived a comprehensive model to describe the dynamics and steady states of a modulated backscatter-free ring cavity laser with a fast gain recovery time. We showed that, unlike slow gain systems with dynamics governed by dissipation, the fast gain supports the expansion of a quantum walk in a synthetic frequency dimension. The expansion continues until the maximum frequency bandwidth limit, set by dispersion and modulation, is reached. Once the system reaches this limit, it stabilizes on the broadest available state in the system that follows a predictable shape given by the nonlinear analytical solution that we derived. Interestingly, when Fabry–Perot fast-gain lasers are strongly modulated, experiments in [33], [47], [65] present instantaneous frequencies that resemble the analytical solutions we have found of a half-sine shape. The appearance of this signature is related to the competition between the cross-steepening nonlinearity caused by spatial hole burning and phase modulation and will be the subject of future studies.

Moreover, we found that detuning from resonance reshapes the state until an abrupt transition occurs, and the state changes its nature to an FM-like comb which directly follows the modulation. We also studied the noise properties of such systems and showed that they outperform their slow gain counterparts, an advantange which we attribute to the liquid state of the light. Although the repetition rate has exquisite locking properties, the jitter in the carrier envelope offset frequency can contribute to substantial optical linewidth broadening. It would be beneficial to study the effects of optically injecting quantum walk comb sources, which incorporate coherent pumping schemes, such as in [66], to reduce the overall optical linewidth of the source. Along with unique acceptance to shaping the spectrum [50], we believe that modulated fast-gain lasers will pave the way to highly controllable, stable and broadband devices for daily applications.

## Appendix A: Derivation of the coupled amplitude and phase equations

Here we derive the coupled amplitude and phase equations. We start with the equation

$$\begin{array}{ll} \dot{E} & =\frac{1}{2}c\left[g_{0}\left(1-\frac{I\left(\eta ,\tau \right)}{I_{\text{sat}}}\right)-\alpha_{w}\right]E+\frac{1}{2}\left(i\beta +g_{c}\right)K^{2}\frac{\partial^{2}E}{\partial \eta^{2}}\\ & \;+i\frac{1}{2}ca_{1}\cos\left(\eta \right)E, \end{array}$$

where we used the copropagating coordinate *η* = *Kz* − ΔΩ*τ*. Due to the instantaneous gain saturation term, we then assume the field follows the following form

$$E=A_{0}\left(1-f_{A}\left(\eta \right)\right)e^{i\phi \left(\eta \right)+i\Delta \omega \tau },$$

where the amplitude is quasi constant *A*_0_ with a small amplitude variation *f*_*A*_, and the state has an overall evolution frequency Δ*ω*. The LHS is then

$$\dot{E}=\left(i\Delta \omega -i\phi^{\prime }\Delta \Omega \right)E+A_{0}f_{A}^{\prime }\Delta \Omega e^{i\phi +i\Delta \omega \tau }.$$

The second derivative of the field in *η* is

$$\begin{array}{ll} \frac{\partial^{2}}{\partial \eta^{2}}E & =\frac{\partial }{\partial \eta }\left(\frac{\partial }{\partial \eta }A_{0}\left(1-f_{A}\right)e^{i\phi +i\Delta \omega \tau }\right)\\ & =A_{0}\frac{\partial }{\partial \eta }\left(-f_{A}^{\prime }e^{i\phi +i\Delta \omega \tau }+\left(1-f_{A}\right)i\phi^{\prime }e^{i\phi +i\Delta \omega \tau }\right)\\ & =A_{0}\left(-f_{A}^{\prime \prime e^{i\phi +i\Delta \omega \tau }}-2f_{A}^{\prime }i\phi^{\prime }e^{i\phi +i\Delta \omega \tau }\right.\\ & \;\left.+\left(1-f_{A}\right)i\phi^{\prime \prime }e^{i\phi +i\Delta \omega \tau }-\left(1-f_{A}\right)\phi^{\prime 2}e^{i\phi +i\Delta \omega \tau }\right). \end{array}$$

We then get

$$\begin{array}{ll} & \left(i\Delta \omega -i\phi^{\prime }\Delta \Omega \right)+\frac{f_{A}^{\prime }}{\left(1-f_{A}\right)}\Delta \Omega \\ & \;=\frac{1}{2}c\left[g_{0}\left(1-\frac{A_{0}^{2}{\left(1-f_{A}\right)}^{2}}{I_{\text{sat}}}\right)-\alpha_{w}\right]\\ & \;\;+\left(iD+G_{c}\right)\left(-\frac{f_{A}^{\prime \prime }}{\left(1-f_{A}\right)}-\frac{2if_{A}^{\prime }}{\left(1-f_{A}\right)}i\phi^{\prime }+i\phi^{\prime \prime }-\phi^{\prime 2}\right)\\ & \;\;+i2Ccos\left(\eta \right). \end{array}$$

We require that the real and imaginary parts will be fulfilled separately

(1) $$\begin{array}{ll} \Delta \omega -\phi^{\prime }\Delta \Omega & =-D\frac{f_{A}^{\prime \prime }}{\left(1-f_{A}\right)}-G_{c}\frac{2if_{A}^{\prime }}{\left(1-f_{A}\right)}\phi^{\prime }+G_{c}\phi^{\prime \prime }\\ & \;-D\phi^{\prime 2}+2Ccos\left(\eta \right), \end{array}$$
(2) $$\begin{array}{ll} \;\frac{f_{A}^{\prime }}{\left(1-f_{A}\right)}\Delta \Omega & =\frac{1}{2}c\left[g_{0}\left(1-\frac{A_{0}^{2}{\left(1-f_{A}\right)}^{2}}{I_{\text{sat}}}\right)-\alpha_{w}\right]\\ & \;-G_{c}\frac{f_{A}^{\prime \prime }}{\left(1-f_{A}\right)}+D\frac{2f_{A}^{\prime }}{\left(1-f_{A}\right)}\phi^{\prime }-D\phi^{\prime \prime }-G_{c}\phi^{\prime 2}. \end{array}$$

## Appendix B: Rationale for neglecting gain curvature

To justify neglecting the term *G*_*c*_ *ϕ*″ in equation Eq. (8), we estimate the values at resonance. The solution for ΔΩ = 0 gives $\phi^{\prime \prime }=\mp \sqrt{\frac{C}{D}}\sin\left(\frac{\eta }{2}\right)$ , and the ratio between the contributions of gain curvature and dispersion are given by

$$\left|\frac{G_{c}\phi^{\prime \prime }}{D\phi^{\prime 2}}\right|=\frac{G_{c}\sqrt{\frac{C}{D}}\sin\left(\frac{\eta }{2}\right)}{D4\frac{C}{D}\cos^{2}\left(\frac{\eta }{2}\right)}=\frac{G_{c}}{4\sqrt{CD}}\frac{\sin\left(\frac{\eta }{2}\right)}{\cos^{2}\left(\frac{\eta }{2}\right)}.$$

As long as $G_{c}\ll 4\sqrt{CD}$ , our approximation is valid, where we can also express this condition as *G*_*c*_ ≪ *Dm*_tot_, where *m*_*tot*_ is the number of populated modes in the steady state at resonance. For realistic parameters, the ratio $\frac{G_{c}}{4\sqrt{CD}}∼\frac{1}{40}$ . Moreover, *G*_*c*_ ∼ *D*, so that as long as the steady state is sufficiently broad, the gain curvature is not playing a significant role in shaping the frequencies. However, at values of *η* = *π*, the $\cos^{2}\left(\frac{\eta }{2}\right)$ drops to zero and the approximation does not hold anymore.

## Appendix C: Considerations for amplitude variation at the near-resonant regime

We start with the equation for the real part of the field

$$\begin{array}{ll} \frac{f_{A}^{\prime }}{\left(1-f_{A}\right)}\Delta \Omega & =\frac{1}{2}c\left[g_{0}\left(1-\frac{A_{0}^{2}{\left(1-f_{A}\right)}^{2}}{I_{\text{sat}}}\right)-\alpha_{w}\right]\\ & \;-G_{c}\frac{f_{A}^{\prime \prime }}{\left(1-f_{A}\right)}+D\frac{2f_{A}^{\prime }}{\left(1-f_{A}\right)}\phi^{\prime }-D\phi^{\prime \prime }-G_{c}\phi^{\prime 2}. \end{array}$$

We consider *G*_*c*_ → 0, therefore the equation is reduced to

$$\begin{array}{ll} f_{A}^{\prime }\Delta \Omega & =\frac{1}{2}c\left[g_{0}\left(1-\frac{A_{0}^{2}{\left(1-\epsilon f_{A}\right)}^{2}}{I_{\text{sat}}}\right)-\alpha_{w}\right]\\ & \;+2Df_{A}^{\prime }\phi^{\prime }-D\phi^{\prime \prime }. \end{array}$$

Taking an order of magnitude for the values of $\phi^{\prime },\phi^{\prime \prime }∼\sqrt{\frac{C}{D}}$ , we can estimate

$$\begin{array}{ll} \epsilon f_{A}^{\prime }\Delta \Omega & =\frac{1}{2}c\left[g_{0}\left(1-\frac{A_{0}^{2}{\left(1-\epsilon f_{A}\right)}^{2}}{I_{\text{sat}}}\right)-\alpha_{w}\right]\\ & \;+2\epsilon f_{A}^{\prime }\sqrt{CD}-\sqrt{CD}. \end{array}$$

Using ΔΩ ≤ Δ_c_ which is the limit in the resonant regime, we can neglect the terms that depend on $f_{A}^{\prime }$ , as $f_{A}^{\prime }\ll 1$ . We finally get

$$D\phi^{\prime \prime }=\frac{1}{2}c\left[g_{0}\left(1-\frac{A_{0}^{2}{\left(1-f_{A}\right)}^{2}}{I_{\text{sat}}}\right)-\alpha_{w}\right].$$

From this formula, we can derive the steady state amplitude

$$\begin{array}{ll} A_{0}\left(1-f_{A}\right) & =\sqrt{I_{s}}\sqrt{1-\frac{2D}{cg_{0}}\phi^{\prime \prime }-\frac{\alpha_{W}}{g_{0}}}\\ & =\sqrt{I_{s}}\sqrt{1-\frac{\alpha_{W}}{g_{0}}}\sqrt{1-\frac{2D}{c\left(g_{0}-\alpha_{W}\right)}\phi^{\prime \prime }}\\ & \approx \sqrt{I_{s}\left(1-\frac{\alpha_{W}}{g_{0}}\right)}\left(1-\frac{D}{c\left(g_{0}-\alpha_{W}\right)}\phi^{\prime \prime }\right). \end{array}$$

So that

$$A_{0}=\sqrt{I_{s}\left(1-\frac{\alpha }{g_{0}}\right)}\;\text{and}\;f_{A}=\frac{D}{c\left(g_{0}-\alpha_{W}\right)}\phi^{\prime \prime },$$

and finally

$$f_{A}\approx \mp \frac{\sqrt{DC}}{c\left(g_{0}-\alpha_{W}\right)}\sin\left(\frac{\eta }{2}\right).$$

## Appendix D: Parameters relationship to physical quantities

To relate the normalized parameters to physical quantities we present in Table A1 a list of physical quantities that corresponds to the system we study in this work.

**Table A1:** Parameters used for simulations.

Name	Symbol	Value
Cavity length	*L*	5 mm
Waveguide losses + output coupling divided by length	*α*_*w*_	7 cm^−1^
Dispersion	*k*_*t*_	1,000 fs^2^/mm
Saturation intensity	*I*_*s*_	8 ⋅ 10^12^ V^2^/m^2^
Coherence lifetime	*T*_2_	50 fs
Light velocity in the cavity	*c*	*c*_0_/*n*, *n* = 3
Gain	*g*_0_	10 cm^−1^
Detuning (angular frequency)	ΔΩ	$\in \left[-2\pi \times 15\;\text{MHz},2\pi \times 15\;\text{MHz}\right]$
Coupling strength	*C*	3 ⋅ 10^8^ rad/s

## Appendix E: Fast-gain operator in the synthetic frequency space

We consider a differential equation with a linear operator local in *z* and the fast-gain nonlinear term

$$i\dot{E}=L_{z}E-g_{0}\frac{I}{I_{s}}E.$$

We use a modal description of the field where $E\left(z,\tau \right)=\sum A_{n}\left(\tau \right)e^{-inKz}$

$$\begin{array}{ll} i\sum {\dot{A}}_{n}\left(\tau \right)e^{-inKz} & =L_{z}\sum A_{n}\left(\tau \right)e^{-inKz}-\frac{g_{0}}{I_{s}}\sum A_{j}\left(\tau \right)\\ & \;\times e^{-ijKz}\sum \overset{*}{\underset{p}{A}}\left(\tau \right)e^{ipKz}\\ & \;\times \sum A_{l}\left(\tau \right)e^{-ilKz}. \end{array}$$

We multiply both side by *e* ^−*im*Kz^, integrate over the cavity and get

$$\begin{array}{ll} i{\dot{A}}_{m}\left(\tau \right) & =\left[L_{z}\left(e^{-imKz}\right)e^{imKz}\right]A_{m}\left(\tau \right)\\ & \;-\int \frac{g_{0}}{I_{s}}\sum A_{j}\left(\tau \right)\overset{*}{\underset{p}{A}}\left(\tau \right)A_{l}\left(\tau \right)e^{i\left(p-j-l+m\right)Kz}dz\\ & =\left[L_{o}\left(e^{-imKz}\right)e^{imKz}\right]A_{m}\left(\tau \right)\\ & \;-\frac{g_{0}}{I_{s}}\underset{j,p,l}{\sum }A_{j}\left(\tau \right)A_{p}^{*}\left(\tau \right)A_{l}\left(\tau \right)\delta_{j+l-p-m}, \end{array}$$

therefore, we can write the nonlinear term of the fast gain as

$$F_{NL,m}=\underset{\mathrm{jpl}}{\sum }\delta_{j+l-m-p}A_{j}A_{l}A_{p}^{*},$$

which is identical to the Kerr term in the modal space, but with an imaginary factor of *i* with respect to the field derivative, making this term nonlinear and non-Hermitian.

## References

[1] Chang L., Liu S., Bowers J. E. (2022). Integrated optical frequency comb technologies. *Nat. Photonics*.

[2] Haus R. (1994). Mobile Fourier-transform infrared spectroscopy monitoring of air pollution. *Appl. Opt.*.

[3] Veerasingam S. (2021). Contributions of Fourier transform infrared spectroscopy in microplastic pollution research: a review. *Crit. Rev. Environ. Sci. Technol.*.

[4] Meyer P. L., Sigrist M. W. (1990). Atmospheric pollution monitoring using CO2‐laser photoacoustic spectroscopy and other techniques. *Rev. Sci. Instrum.*.

[5] Elsaesser A. (2020). SpectroCube: a European 6U nanosatellite spectroscopy platform for astrobiology and astrochemistry. *Acta Astronaut.*.

[6] Marin-Palomo P. (2017). Microresonator-based solitons for massively parallel coherent optical communications. *Nature*.

[7] Corcoran B. (2020). Ultra-dense optical data transmission over standard fibre with a single chip source. *Nat. Commun.*.

[8] Hu H., Oxenløwe L. K. (2021). Chip-based optical frequency combs for high-capacity optical communications. *Nanophotonics*.

[9] Lukashchuk A., Riemensberger J., Tusnin A., Liu J., Kippenberg T. J. (2023). Chaotic microcomb-based parallel ranging. *Nat. Photonics*.

[10] Lukashchuk A. (2024). Photonic-electronic integrated circuit-based coherent LiDAR engine. *Nat. Commun.*.

[11] Gaeta A. L., Lipson M., Kippenberg T. J. (2019). Photonic-chip-based frequency combs. *Nat. Photonics*.

[12] Khurgin J. B., Clerici M., Kinsey N. (2021). Fast and slow nonlinearities in epsilon-near-zero materials. *Laser Photonics Rev.*.

[13] Kippenberg T. J., Gaeta A. L., Lipson M., Gorodetsky M. L. (2018). Dissipative Kerr solitons in optical microresonators. *Science*.

[14] Herr T. (2012). Universal formation dynamics and noise of Kerr-frequency combs in microresonators. *Nat. Photonics*.

[15] Bowers J. E., Morton P. A., Mar A., Corzine S. W. (1989). Actively mode-locked semiconductor lasers. *IEEE J. Quantum Electron.*.

[16] Hugi A., Villares G., Blaser S., Liu H. C., Faist J. (2012). Mid-infrared frequency comb based on a quantum cascade laser. *Nature*.

[17] Burghoff D. (2014). Terahertz laser frequency combs. *Nat. Photonics*.

[18] Sato K. (2003). Optical pulse generation using fabry-Perot lasers under continuous-wave operation. *IEEE J. Sel. Top. Quantum Electron.*.

[19] Hillbrand J. In-phase and anti-phase synchronization in a laser frequency comb. *Phys. Rev. Lett*..

[20] Rosales R. (2012). High performance mode locking characteristics of single section quantum dash lasers. *Opt. Express*.

[21] Joshi S. (2014). Quantum dash based single section mode locked lasers for photonic integrated circuits. *Opt. Express*.

[22] Dong B., Dumont M., Terra O., Wang H., Netherton A., Bowers J. E. (2023). Broadband quantum-dot frequency-modulated comb laser. *Light: Sci. Appl.*.

[23] Aranson I. S., Kramer L. (2002). The world of the complex Ginzburg-Landau equation. *Rev. Mod. Phys.*.

[24] Carusotto I., Ciuti C. (2013). Quantum fluids of light. *Rev. Mod. Phys.*.

[25] Richard M., Kasprzak J., Romestain R., André R., Dang L. S. (2005). Spontaneous coherent phase transition of polaritons in CdTe microcavities. *Phys. Rev. Lett*..

[26] Schneider C. (2013). An electrically pumped polariton laser. *Nature*.

[27] Scheuer J., Orenstein M. (1999). Optical vortices crystals: spontaneous generation in nonlinear semiconductor microcavities. *Science*.

[28] Piccardo M., Capasso F. Laser frequency combs with fast gain recovery: physics and applications. *Laser Photonics Rev.*.

[29] Dikopoltsev A., Heckelmann I., Bertrand M., Beck M., Scalari G., Zilberberg O., Faist J. (2025). Collective quench dynamics of active photonic lattices in synthetic dimensions,. *Nat. Phys.*.

[30] Khurgin J. B., Dikmelik Y., Hugi A., Faist J. (2014). Coherent frequency combs produced by self frequency modulation in quantum cascade lasers. *Appl. Phys. Lett*..

[31] Haus H. A. (2000). Mode-locking of lasers. *IEEE J. Sel. Top. Quantum Electron.*.

[32] DeMaria A. J., Stetser D. A., Heynau H. (1966). Self mode‐locking of lasers with saturable absorbers. *Appl. Phys. Lett.*.

[33] Senica U. Frequency-modulated combs via field-enhancing tapered waveguides. *Laser Photonics Rev.*.

[34] Täschler P. (2021). Femtosecond pulses from a mid-infrared quantum cascade laser. *Nat. Photonics*.

[35] Singleton M., Jouy P., Beck M., Faist J. (2018). Evidence of linear chirp in mid-infrared quantum cascade lasers. *Optica*.

[36] Opačak N., Schwarz B. (2019). Theory of frequency-modulated combs in lasers with spatial hole burning, dispersion, and Kerr nonlinearity. *Phys. Rev. Lett.*.

[37] Burghoff D. (2020). Unraveling the origin of frequency modulated combs using active cavity mean-field theory. *Optica*.

[38] Humbard L., Burghoff D. (2022). Analytical theory of frequency-modulated combs: generalized mean-field theory, complex cavities, and harmonic states. *Opt. Express*.

[39] Piccardo M. (2020). Frequency combs induced by phase turbulence. *Nature*.

[40] Heckelmann I., Bertrand M., Dikopoltsev A., Beck M., Scalari G., Faist J. (2023). Quantum walk comb in a fast gain laser. *Science*.

[41] Marzban B., Miller L., Dikopoltsev A., Bertrand M., Scalari G., Faist J. (2024). *A Quantum Walk Comb Source at Telecommunication Wavelengths*.

[42] Seitner L. Backscattering-induced dissipative solitons in ring quantum cascade lasers. *Phys. Rev. Lett*..

[43] Revin D. G., Hemingway M., Wang Y., Cockburn J. W., Belyanin A. (2016). Active mode locking of quantum cascade lasers in an external ring cavity. *Nat. Commun.*.

[44] Hillbrand J. (2020). Mode-locked short pulses from an 8 μm wavelength semiconductor laser. *Nat. Commun.*.

[45] Opačak N., Cin S. D., Hillbrand J., Schwarz B. (2021). Frequency comb generation by bloch gain induced giant Kerr nonlinearity. *Phys. Rev. Lett.*.

[46] Osinski M., Buus J. (1987). Linewidth broadening factor in semiconductor lasers--An overview. *IEEE J. Quantum Electron.*.

[47] Opačak N., Schneider B., Faist J., Schwarz B. (2024). Impact of higher-order dispersion on frequency-modulated combs. *Opt. Lett.*.

[48] Haus H. (1975). A theory of forced mode locking. *IEEE J. Quantum Electron.*.

[49] Yuan L., Lin Q., Xiao M., Fan S. (2018). Synthetic dimension in photonics. *Optica*.

[50] Piciocchi D., Dikopoltsev A., Heckelmann I., Beck M., Scalari G., Faist J. (2025). Frequency comb shaping through staggered phase flux in fast gain lasers. ..

[51] Schreiber A. (2010). Photons walking the line: a quantum walk with adjustable coin operations. *Phys. Rev. Lett.*.

[52] Gräfe M., Heilmann R., Lebugle M., Guzman-Silva D., Perez-Leija A., Szameit A. (2016). Integrated photonic quantum walks. *J. Opt.*.

[53] Peruzzo A. (2010). Quantum walks of correlated photons. *Science*.

[54] Broome M. A., Fedrizzi A., Lanyon B. P., Kassal I., Aspuru-Guzik A., White A. G. (2010). Discrete single-photon quantum walks with tunable decoherence. *Phys. Rev. Lett.*.

[55] Karski M. (2009). Quantum walk in position space with single optically trapped atoms. *Science*.

[56] Meng B. (2020). Mid-infrared frequency comb from a ring quantum cascade laser. *Optica*.

[57] Meng B., Singleton M., Hillbrand J., Franckié M., Beck M., Faist J. (2022). Dissipative Kerr solitons in semiconductor ring lasers. *Nat. Photonics*.

[58] Childs A. M., Cleve R., Deotto E., Farhi E., Gutmann S., Spielman D. A. (2003). *Proceedings of the thirty-fifth annual ACM symposium on Theory of computing*.

[59] Mülken O., Blumen A. (2011). Continuous-time quantum walks: Models for coherent transport on complex networks. *Physics Reports*.

[60] Weill R., Levit B., Bekker A., Gat O., Fischer B. (2010). Laser light condensate: experimental demonstration of light-mode condensation in actively mode locked laser. *Opt. Express*.

[61] Amelio I., Chiocchetta A., Carusotto I. (2024). Kardar-Parisi-Zhang universality in the coherence time of nonequilibrium one-dimensional quasicondensates. *Phys. Rev. E*.

[62] Amelio I., Carusotto I. Theory of the coherence of topological lasers. *Phys. Rev. X*.

[63] Chiocchetta A., Carusotto I. (2013). Non-equilibrium quasi-condensates in reduced dimensions. *Europhys. Lett*..

[64] Khurgin J. B., Henry N., Burghoff D., Hu Q. (2018). Linewidth of the laser optical frequency comb with arbitrary temporal profile. *Appl. Phys. Lett.*.

[65] Schneider B. Controlling quantum cascade laser optical frequency combs through microwave injection. *Laser Photonics Rev.*.

[66] Hillbrand J., Andrews A. M., Detz H., Strasser G., Schwarz B. (2019). Coherent injection locking of quantum cascade laser frequency combs. *Nat. Photonics*.

